# The COVID-19 pandemic and Asian American employment

**DOI:** 10.1007/s00181-022-02306-5

**Published:** 2022-10-29

**Authors:** Bo E. Honoré, Luojia Hu

**Affiliations:** 1 Department of Economics, Julis Romo Rabinowitz Building, Princeton, NJ 08544 USA; 2 Economic Research Department, Federal Reserve Bank of Chicago, 230 S. La Salle Street, Chicago, IL 60604 USA

**Keywords:** Employment, Pandemic, Asian Americans, Racial disparity, J21, J70, J71

## Abstract

Recent studies have documented the disparate impact of the COVID-19 pandemic on labor market outcomes for different racial groups. This paper adds to this literature by documenting that the employment of Asian Americans—in particular those with no college education—has been especially hard hit by the economic crisis associated with the onset of the pandemic. This can only partly be explained by differences in demographics, local market conditions, and job characteristics, and it also cannot be entirely explained by possible different selection into education levels across ethnic groups. The burden on Asian Americans is primarily borne by those who are not US-born.

**Supplementary Information:**

The online version contains supplementary material available at 10.1007/s00181-022-02306-5.

## Introduction

A number of papers have documented the disparate impact of the COVID-19 pandemic on labor market outcomes across racial groups. This includes (Bartik et al. 2020; Cortes and Forsythe 2020; Dam et al. 2021), and Lee et al. (2021). With the exception of Lee et al. (2021), this literature has primarily focused on Blacks and Hispanics. At the same time, there is evidence that the health of Asian Americans was disproportionately impacted by the pandemic. See, for example, Marcello et al. (2020). The contribution of this paper is to study the disparity across a broader group of ethnicities which includes Asian Americans. We find that Asian Americans were also disproportionately hard hit by the onset of the pandemic in terms of employment, though they recovered more quickly than other minority groups.

**Fig. 1:**
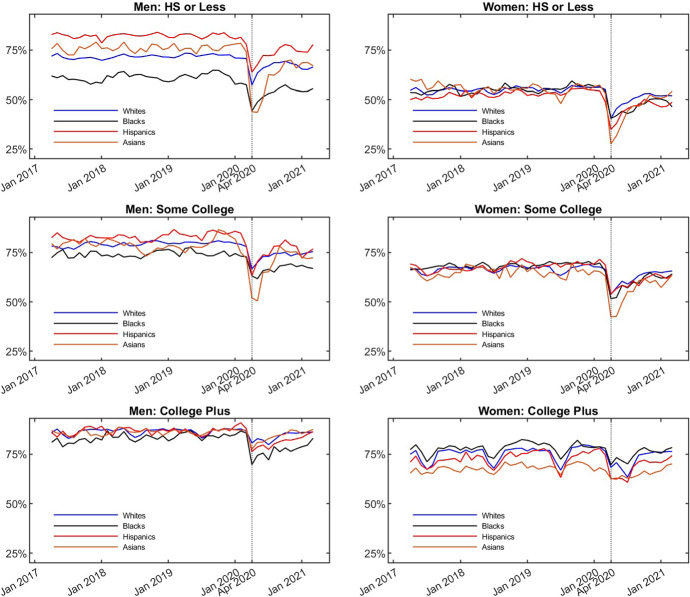
Fraction Working by Group. The figure plots the fraction of observations that report working in a given month for each combination of gender, education and ethnicity group. The data is from IPUMS CPS and covers individuals aged 25-65 over the period between April 2017 and March 2021. The fractions are calculated using sampling weights

The simplest facts are displayed in Fig. 1. The figure shows that the employment of minority groups (Blacks, Hispanics, and Asian Americans) was more negatively impacted by the pandemic than the employment of Whites. The decline in employment was sharpest at the start of the pandemic. For example, the employment rates dropped by 9 and 11 percentage points for men and for women, respectively, from the first to the second quarter of 2020, and recovered by 5.8 and 3.4 percentage points from the second to the fourth quarter. While all groups experienced a fall in employment, the effect was most dramatic for low-educated Asian Americans. For example, Asian American men with a high school degree or less experienced a 31 percentage point drop in employment in the second quarter. The corresponding drop for Asian American women with a high school degree or less was 23 percentage points.^1^

Prior to this paper, the empirical patterns regarding Asian American employment seem to have been largely overlooked. The report by Mar and Ong (2020) is a notable exception. They report aggregate statistics to compare the unemployment rate for Asian Americans and Whites before and after the onset of the crisis. The contribution of this paper is to use micro data to investigate the extent to which the different employment patterns across the groups (especially Asian Americans) reported above can be explained by differences in demographics, local labor market conditions, and job characteristics at the individual level. In order to allow for the possibility that the labor market treatment of Asian Americans is related to their appearance, we initially disaggregate the group of Asian Americans into those of East Asian descent, those of South-East Asian descent, and the remaining group of Asians, and compare those groups to Whites, Blacks, and Hispanics.

We argue that the differences in the effect of the pandemic on employment cannot be explained by differences in other demographic characteristics or local labor market conditions. We document these findings in Sect. 3 below.

In Sect. 4, we focus on transitions in and out of employment. We find that the large differential change in the probability of employment across ethnicity is mainly driven by differences in the probability of remaining employed and less by differences in the probability of gaining employment. Studying transitions also allows us to control for job characteristics such as industry and occupation for an individual. This is potentially important because some industries were considered “essential” at the start of the pandemic and the employment in those industries was impacted very differently from that in those considered non-essential. At the same time, it is easier to work from home in certain occupations than in others. Since workers of different ethnicities tend to work in different types of jobs, it is important to control for industry or occupation when estimating the differential impact on employment across different ethnicities. We find that differences in occupation and industry can explain up to half of the differential between Asians and Whites in the probability of remaining employed. For example, after controlling for other demographics, local labor market conditions, and occupation, low-educated East and South-East Asian American men experienced a 15 percentage point higher probability of losing their job in the second quarter of 2020 than comparable Whites. Without controlling for occupation, the corresponding number is 22 percentage points.

In Sect. 5, we explore various explanations for the remaining large differences between Asian Americans and other groups. One potential explanation is that the well-documented increase in anti-Asian sentiments in the United States around the onset of the pandemic has led to increased labor market discrimination against Asian Americans. Another possible difference between groups is household structure. For example, if Asian Americans are more likely to live in multi-generational households, then concerns for the health of older family members might make them more likely to withdraw from the labor market in response to the pandemic. Alternatively, individuals with a young child might be constrained by closures of schools and daycare centers. Finally, many Asian American are immigrants. This raises the question of whether the larger effect on Asian Americans is driven by their country of birth. A number of interesting findings emerge from the analysis in this section. First, we find no evidence that the differential employment impact on Asians is larger in occupations that are characterized by more personal interactions. We also do not find evidence that Asian Americans are especially hard hit in states with larger anti-Asian sentiments. Thirdly, the patterns do not seem to be driven by individuals who stop working due to health concerns about elderly household members or due to the presence of a young child. Finally, we find that the impact on Asian Americans is largely borne by immigrants.

The effect of the pandemic on employment differs significantly across education groups as well as ethnicity. At the same time, Asian Americans tend to be higher educated than other groups. This raises the question of whether the especially large effect on low-educated Asian Americans is driven by different selection into educational attainment across ethnic groups. In Sect. 6, we find that this cannot fully explain the differences in the effect of the pandemic on employment across ethnic groups. Section 7 concludes the paper.

## Data and summary statistics

For the analysis in this paper, we use the Current Population Survey (CPS) Basic Monthly micro data from April of 2017 to March of 2021. This covers three full years before the approximate onset of the pandemic and the first year after the onset. The data is downloaded from https://www.ipums.org/ (Flood et al. 2021). The monthly CPS has a panel design. Households are interviewed for four consecutive months, then not interviewed for eight months, and finally interviewed for four more consecutive months. In the analysis below, this allows us to consider transitions in and out of employment. The data structure also suggests that it will be important to consider correlations between observations in the sample, since multiple members of the same household may appear in the sample and the same individual will be interviewed in multiple months. Except where stated, we account for this potential correlation by reporting standard errors that are clustered at the household level.

To present summary statistics for the variables that are most important for our analysis, we disaggregate the data by gender, educational attainment, and ethnicity, where the term “ethnicity” is defined based on both what is usually called race and what is usually called ethnicity. Specifically, we use the IPUMS CPS variables^2^ “race” and “hispan” to define ethnicity: “Whites” are the individuals who report their race as white and report to be non-hispanic; “Blacks” are the individuals who report their race as black; and “Hispanics” are the individuals who report their race to be white and report to not be non-hispanic. The group “Asians” is composed of those who report their race as “asian only”, “hawaiian/pacific islander only”, “white-asian”, or “black-asian”. Other races and ethnicities are dropped from the analysis.

Since Asian Americans are a very heterogeneous group, we divide Asians into a group labeled “AsianEast” and a group labeled “AsianOther”. The group AsianEast is composed of two subsets, East Asians and South-East Asians. East Asians is the subset of Asians that are either born, or have a parent born, in Japan, North Korea, Taiwan, Hong Kong, South Korea, Mongolia, China or Macau. South-East Asians is the subset of Asians that are not East Asians, and are either born, or have a parent born, in Brunei, Cambodia, Indonesia, Laos, Malaysia, Burma, Myanmar, Philippines, Singapore, Thailand, Timor Leste, or Vietnam. All other Asians are labelled AsianOther.

**Table 1:** Summary Statistics

	Men HS or less	Men Some Col.	Men College+	Women HS or less	Women Some Col.	Women College+
	Sample Size					
Whites	344,216	266,045	375,157	286,784	298,885	444,873
Blacks	65,405	38,491	32,961	69,743	55,347	51,009
Hispanics	111,508	37,867	31,461	105,502	46,442	41,804
AsianEast	14,090	9,926	26,225	17,769	11,741	34,551
AsianOther	9,453	6,300	26,361	9,550	6,777	25,246
	Sample Size - Unique Individuals					
Whites	79,350	61,223	82,337	66,832	68,524	96,705
Blacks	16,864	10,008	8,372	17,897	14,244	12,647
Hispanics	26,543	9,688	8,023	24,799	11,671	10,406
AsianEast	3,432	2,472	6,174	4,201	2,967	7,977
AsianOther	2,591	1,740	6,339	2,549	1,835	6,055
	Fraction Working					
Whites	0.703	0.778	0.859	0.538	0.656	0.747
Blacks	0.591	0.723	0.818	0.530	0.662	0.772
Hispanics	0.800	0.809	0.853	0.509	0.659	0.717
AsianEast	0.721	0.765	0.834	0.570	0.642	0.706
AsianOther	0.733	0.762	0.880	0.457	0.606	0.625
	Age					
Whites	46.59	45.61	44.99	48.05	46.44	44.34
Blacks	44.29	42.85	42.89	44.95	43.49	43.23
Hispanics	42.54	40.74	42.05	43.66	41.13	41.25
AsianEast	46.32	42.80	42.28	48.26	44.11	42.44
AsianOther	43.88	41.56	40.30	44.66	41.49	39.66
	Fraction with Children					
Whites	0.376	0.413	0.451	0.434	0.482	0.485
Blacks	0.330	0.372	0.419	0.521	0.545	0.495
Hispanics	0.561	0.477	0.472	0.702	0.618	0.544
AsianEast	0.553	0.443	0.464	0.610	0.547	0.500
AsianOther	0.561	0.491	0.547	0.671	0.595	0.606
	Fraction with Children Under 5					
Whites	0.090	0.115	0.140	0.093	0.116	0.145
Blacks	0.094	0.113	0.138	0.130	0.142	0.125
Hispanics	0.154	0.155	0.142	0.164	0.173	0.165
AsianEast	0.106	0.100	0.147	0.097	0.125	0.148
AsianOther	0.147	0.160	0.207	0.145	0.173	0.220
	Fraction Married					
Whites	0.539	0.597	0.678	0.575	0.600	0.666
Blacks	0.355	0.408	0.516	0.282	0.302	0.415
Hispanics	0.581	0.525	0.579	0.572	0.514	0.583
AsianEast	0.654	0.557	0.633	0.681	0.625	0.663
AsianOther	0.621	0.586	0.716	0.657	0.587	0.765

The table shows the fraction of individuals in each group that report to be “at work” in a given month. The data is from IPUMS CPS and covers individuals aged 25–65 over the period between April 2017 and March 2021. The averages are calculated using sampling weights

The first panel of Table 1 shows the total sample size for each combination of gender, educational attainment, and ethnicity, while the second shows the number of unique individuals in each group. Since our goal is to study the impact of the pandemic on employment, we restrict the data to individuals aged 25–65. This will reduce the impact of education and retirement decisions. The sample size for each group varies between 6, 300 and 444, 873. The two groups of Asians are the smallest, especially among those without a college degree. The third panel displays the sample fraction working for each group over the sample period. Our data shows some familiar patterns: for example, among men, Hispanics are more likely to work than others, while Blacks at the lower end of the education distribution are less likely to work.

The last four panels of Table 1 show sample means for age, the fractions of observations that have a child, the fractions of observations that have a child under age 5, and the fractions that report being married. There are notable differences in these averages across the groups, which suggests that it might be important to control for these variables when investigating the disparate effect of the pandemic on the probability of working.

## The probability of working

In this section, we first present some simple facts about the differences in employment outcomes across gender, education and ethnic groups before and during the pandemic. We then turn to regression models that control for other observable factors.

### Summary statistics for the probability of working

The simplest facts regarding employment were already displayed in Fig. 1. That figure displays the fraction of each group that reports working between April 2017 and March 2021.^3^ Overall, the employment rate for men declined by 9 percentage points in the second quarter of 2020 relative to the previous quarter. The minority groups, Blacks, Hispanics, and Asians, suffered steeper declines ranging between 10 and 14 percentage points. By the fourth quarter of 2020, the decline in employment for Asian Americans had recovered to close to that of Whites (at approximately 2.5 percentage point lower than the first quarter of 2020), while the negative effects on Blacks and Hispanics persisted at more than 4 percentage points below their employment rate in the first quarter of 2020.

The most striking feature of Fig. 1 is a dramatic decline in employment for Asian Americans with a high school degree or less. In the first quarter of 2020, 77% of Asian men in this group reported working. In the second quarter, the rate fell by 31 percentage points to 46%. By contrast, the changes for comparable Whites, Blacks and Hispanics were approximately 9, 10 and 12 percentage points, respectively. The patterns in the changes for women with a high school degree or less are similar to those for men^4^.

### Controlling for other characteristics

In this subsection, we present the results from estimating linear probability models for the probability of working. We use data from April 2017 to March 2021 (covering 36 months before the onset of the pandemic and 12 months after). Our main goal is to document how working depends on ethnicity before and during the pandemic. The variables of interest will therefore be interactions between ethnicity dummies and COVID periods, starting with the second quarter of 2020 (the pandemic quarters). Below, and in the tables, we refer to the last three quarters of 2020 and the first quarter of 2021 as “Cr2”, “Cr3”, “Cr4”, and “Cr5”.

Whether someone is working is likely to be influenced by the demographic characteristics of the individual. Therefore, for each combination of gender and education group, we estimate a linear probability model for working that controls for age, age squared, marital status, presence of children, presence of children under the age of 5, and interactions between indicators of state of residence and each of the pandemic months starting in April of 2020. The latter controls for geographic variation in the impact of the pandemic on the local economy. To allow for the possibility that the seasonal effects differ by group, we also include interactions between ethnicity and indicators of the four calendar quarters, as well as interactions between ethnicity and each pandemic quarter starting with the second quarter of 2020. In summary, we estimate models of the type

1 $$\begin{aligned} y_{it}=x_{it}^{\prime }\beta +\lambda _{State_{it}, Month_t}+\gamma _{Ethnicity_{i},Quarter_{t}}+\delta _{Ethnicity_{i}, Cr_{t}}+error_{it} \end{aligned}$$

where $y_{it}$ is a dummy variable for working, $x_{it}$ is a set of controls, ${State_it}$ is the state of residence, ${Month_t}$ is a variable for each of the 48 months in the sample, $Ethnicity_{i}$ is the ethnicity of individual *i*, ${Quarter_t}$ is a set of dummy variables for the four calendar quarters, and $Cr_{t}$ is the set of dummy variables corresponding to each crisis quarter. The parameters of interest are the coefficients on the interactions between the ethnicity variables and pandemic quarters (i.e., the $\delta$’s).

The estimated coefficients for the key parameters of interest are reported in Table 2. The results in Table 2 are most striking for individuals with a high school degree or less (the first and fourth columns). Generally speaking, Asian Americans in this group were much harder hit by the pandemic than any other group. This is especially true in the second quarter of 2020, and particularly for East and South East Asians. Controlling for demographics, East Asian men and women have an estimated additional 27 and 19 percentage point drop in the probability of working in the second quarter relative to their white counterparts^5^. The point estimates for the corresponding drop in the probability of working between the first and third quarter were approximately 14 and 11 percentage points. This pattern also holds for South East Asians, although to a lesser degree. The differentials between Whites and Blacks or Hispanics are generally much smaller.

**Table 2:** Linear Probability Model for the Probability of Working with Controls for Demographics and Time-varying Labor Market Conditions

	Men HS or less	Men Some Coll.	Men College+	Women HS or less	Women Some Coll.	Women College+
Black*Cr2	−0.042**	−0.028	−0.054***	−0.023	−0.052***	−0.003
	(0.017)	(0.020)	(0.018)	(0.017)	(0.018)	(0.015)
Hispanic*Cr2	−0.046***	−0.051***	−0.023	−0.030**	−0.020	−0.030*
	(0.013)	(0.019)	(0.017)	(0.015)	(0.019)	(0.018)
AsianE*Cr2	−0.270***	−0.155**	−0.009	−0.194***	−0.139**	0.011
	(0.048)	(0.063)	(0.021)	(0.040)	(0.058)	(0.026)
AsianSE*Cr2	−0.177***	−0.131***	−0.040	−0.226***	−0.104**	0.003
	(0.041)	(0.044)	(0.031)	(0.036)	(0.047)	(0.028)
AsianOther*Cr2	−0.153***	−0.101**	0.003	0.049	−0.069	0.058***
	(0.038)	(0.044)	(0.016)	(0.039)	(0.050)	(0.022)
Black*Cr3	−0.047***	−0.020	−0.041**	−0.067***	−0.055***	0.007
	(0.016)	(0.020)	(0.017)	(0.016)	(0.017)	(0.014)
Hispanic*Cr3	−0.038***	−0.005	−0.024	−0.009	−0.036*	0.001
	(0.012)	(0.017)	(0.016)	(0.015)	(0.019)	(0.016)
AsianE*Cr3	−0.143***	0.043	0.029	−0.107**	−0.077	0.033
	(0.053)	(0.062)	(0.021)	(0.042)	(0.057)	(0.024)
AsianSE*Cr3	−0.064	−0.016	−0.019	−0.044	0.007	−0.033
	(0.040)	(0.043)	(0.031)	(0.039)	(0.044)	(0.027)
AsianOther*Cr3	−0.032	−0.022	0.030**	0.074*	0.012	−0.012
	(0.038)	(0.045)	(0.015)	(0.041)	(0.056)	(0.023)
Black*Cr4	−0.020	0.003	−0.040***	−0.039**	−0.027*	−0.005
	(0.015)	(0.017)	(0.016)	(0.016)	(0.016)	(0.013)
Hispanic*Cr4	−0.018	0.025	−0.029**	−0.012	−0.039**	−0.004
	(0.011)	(0.016)	(0.014)	(0.014)	(0.017)	(0.016)
AsianE*Cr4	−0.104*	0.003	0.014	−0.062	−0.022	0.017
	(0.054)	(0.056)	(0.020)	(0.046)	(0.056)	(0.024)
AsianSE*Cr4	0.002	0.052	0.005	0.025	−0.071	−0.089***
	(0.031)	(0.038)	(0.025)	(0.035)	(0.044)	(0.026)
AsianOther*Cr4	−0.031	0.026	0.039***	0.011	0.052	0.021
	(0.035)	(0.044)	(0.014)	(0.039)	(0.044)	(0.022)
Black*Cr5	−0.002	−0.025	−0.027*	−0.023	−0.034**	−0.010
	(0.016)	(0.018)	(0.015)	(0.017)	(0.017)	(0.014)
Hispanic*Cr5	0.003	−0.041**	−0.009	−0.015	−0.020	−0.011
	(0.011)	(0.017)	(0.014)	(0.014)	(0.018)	(0.016)
AsianE*Cr5	−0.079	−0.102*	−0.001	−0.078	−0.030	0.018
	(0.051)	(0.062)	(0.021)	(0.049)	(0.054)	(0.023)
AsianSE*Cr5	0.029	−0.025	0.024	0.052	0.012	−0.003
	(0.031)	(0.036)	(0.023)	(0.036)	(0.039)	(0.026)
AsianOther*Cr5	−0.039	0.017	0.029**	0.033	−0.049	0.040*
	(0.036)	(0.045)	(0.014)	(0.040)	(0.043)	(0.022)
Observations	544,672	358,629	492,165	489,348	419,192	597,483

The dependent variable is working. The control variables not reported are: age, age$^2$, marital status, presence of a child, presence of a child under 5, interactions between calendar quarter and ethnicity as well as their main effects, and fixed effects for each combination of state and month starting in April 2020. Cr2, Cr3, Cr4, and Cr5 refer to the second quarter of 2020 through the first quarter of 2021. The data is from IPUMS CPS and covers individuals aged 25–65 over the period between April 2017 and March 2021. Robust standard errors are clustered at the household level, and the estimation uses sampling weights

The results for the top end of the educational attainment distribution (columns 3 and 6 of Table 2) are very different. For men with a college degree or more, the decline in employment is similar across ethnic groups, with the exception that Blacks suffered a larger decline than the other groups. Among women in this education group, individuals in the group AsianOther has a smaller employment drop than others. The magnitudes of the differences between ethnic groups among the highly educated are dwarfed, however, by the differences for the lower end of the education distribution.

Since the largest differential effects of the pandemic by ethnicity were in the first two quarters of the pandemic (“Cr2” and “Cr3”), we only report estimates associated with those two quarters in the remainder of the paper.

It is clear from Table 2 that the magnitudes of the estimated effects for East Asians and South East Asians are quite different. On the other hand, the overall patterns are similar. Moreover, some of the estimates are noisy when we restrict estimation to the subsample of individuals with a high school degree or less. Table 3 presents the results for the same model as in Table 2, but with East Asians and South East Asians merged into one group, “AsianEast”. As expected, the overall pattern is the same, with the point estimates for AsianEast of the same magnitude as the point estimates for the dis-aggregated East and South East Asians. Also as expected, the coefficients on AsianEast are more precisely estimated than when this group is dis-aggregated. For most of the rest of the paper, we therefore aggregate East and South East Asian Americans into one group.

**Table 3:** The Probability of Working with Controls for Demographics and Time-varying Labor Market Conditions, Combining East and South East Asians

	Men HS or less	Men Some Coll.	Men College+	Women HS or less	Women Some Coll.	Women College+
Black*Cr2	−0.042**	−0.028	−0.055***	−0.023	−0.052***	−0.003
	(0.017)	(0.020)	(0.018)	(0.017)	(0.018)	(0.015)
Hispanic*Cr2	−0.046***	−0.052***	−0.023	-0.030**	−0.020	−0.030*
	(0.013)	(0.019)	(0.017)	(0.015)	(0.019)	(0.018)
AsianEast*Cr2	−0.213***	−0.139***	−0.020	−0.212***	−0.111***	0.007
	(0.032)	(0.037)	(0.018)	(0.028)	(0.038)	(0.020)
AsianOther*Cr2	−0.153***	−0.102**	0.003	0.050	−0.069	0.058***
	(0.038)	(0.044)	(0.016)	(0.039)	(0.050)	(0.022)
Black*Cr3	−0.047***	−0.020	−0.041**	−0.067***	−0.055***	0.007
	(0.016)	(0.020)	(0.017)	(0.016)	(0.017)	(0.014)
Hispanic*Cr3	−0.039***	−0.005	−0.024	−0.009	−0.036*	0.001
	(0.012)	(0.017)	(0.016)	(0.015)	(0.019)	(0.016)
AsianEast*Cr3	−0.095***	0.006	0.013	−0.077**	−0.022	0.005
	(0.033)	(0.036)	(0.018)	(0.030)	(0.036)	(0.019)
AsianOther*Cr3	−0.033	−0.023	0.030**	0.075*	0.013	−0.012
	(0.038)	(0.045)	(0.015)	(0.041)	(0.056)	(0.023)
Observations	544,672	358,629	492,165	489,348	419,192	597,483

The dependent variable is working. The control variables not reported are: age, age$^2$, marital status, presence of a child, presence of a child under 5, interactions between calendar quarter and ethnicity as well as their main effects, interactions between ethnicity and Cr4 and Cr5, and fixed effects for each combination of state and month starting in April 2020. Cr2, Cr3, Cr4, and Cr5 refer to the second quarter of 2020 through the first quarter of 2021. The data is from IPUMS CPS and covers individuals aged 25-65 over the period between April 2017 and March 2021. Robust standard errors are clustered at the household level, and the estimation uses sampling weights

## Transition in and out of employment

The analysis in Sect. 3 focused on the probability of working. We now turn to the probability of working conditional on whether the individual worked in the previous month, which is made possible by the panel design of the CPS. There are two distinct motivations for focusing on transitions.

The first motivation for focusing on transitions is that it is economically interesting to know whether the large decline in the probability of working for Asian Americans is driven by the probability that the employed stopped working or by the probability that those not employed started working.

**Fig. 2:**
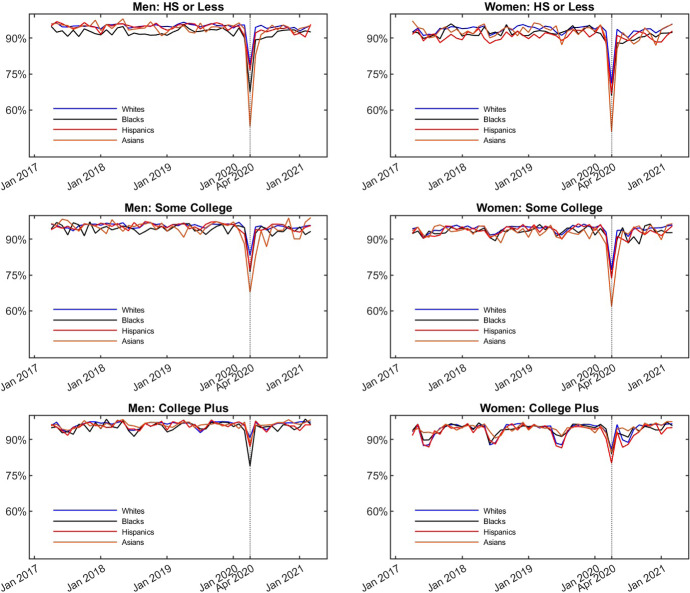
Fraction Remaining at Work by Group. The figure plots the fraction of observations who worked the previous month that also report working in the current month for each combination of gender, education and ethnicity group. The data is from IPUMS CPS and covers individuals aged 25-65 over the period between April 2017 and March 2021. The fractions are calculated using sampling weights

The basic findings regarding month-to-month transitions out of employment are presented in Fig. 2, which displays the fraction of individuals working in the previous month, who are also working in the current month for Whites, Blacks, Hispanics and Asians (combined across all sub-groups). The figure shows a large drop in the probability of remaining employed in April of 2020 for all groups. The decline is larger for those without a college degree and especially large for Asian Americans.

The corresponding figures for the monthly probability of being employed condition on not being employed in previous month are based on much smaller sample sizes and therefore too noisy to be informative. We therefore turn to regressions. We first estimate the econometric model for working from Sect. 3 separately for the samples of individuals who were or were not working in the previous month. The results are presented in Tables 4 and 5.

**Table 4:** The Probability of Remaining Employed with Controls for Demographics and Time-varying Labor Market Conditions

	Men HS or less	Men Some Coll.	Men College+	Women HS or less	Women Some Coll.	Women College+
Black*Cr2	−0.055***	−0.034**	−0.045***	−0.032*	−0.011	−0.016
	(0.015)	(0.015)	(0.014)	(0.017)	(0.014)	(0.011)
Hispanic*Cr2	−0.030***	−0.021	−0.009	−0.014	−0.031**	−0.035***
	(0.010)	(0.013)	(0.010)	(0.015)	(0.015)	(0.013)
AsianEast*Cr2	−0.219***	−0.091***	−0.024*	−0.195***	−0.089**	−0.021*
	(0.037)	(0.034)	(0.012)	(0.040)	(0.037)	(0.013)
AsianOther*Cr2	−0.083**	−0.094**	−0.003	−0.025	−0.108**	0.014
	(0.037)	(0.039)	(0.009)	(0.040)	(0.049)	(0.013)
Black*Cr3	−0.005	−0.002	0.001	−0.022	−0.042***	−0.005
	(0.012)	(0.012)	(0.011)	(0.014)	(0.014)	(0.010)
Hispanic*Cr3	−0.006	0.005	0.004	0.009	−0.000	−0.020
	(0.008)	(0.010)	(0.010)	(0.012)	(0.012)	(0.012)
AsianEast*Cr3	−0.003	−0.043*	−0.003	−0.054**	−0.001	−0.002
	(0.019)	(0.024)	(0.009)	(0.026)	(0.021)	(0.011)
AsianOther*Cr3	0.011	−0.010	0.011	0.052	0.073***	0.006
	(0.024)	(0.029)	(0.008)	(0.039)	(0.021)	(0.014)
Observations	268,219	195,225	295,552	180,794	193,398	311,497

The dependent variable is working, and the sample is restricted to those who had a job in the previous month. The control variables not reported are: age, age$^2$, marital status, presence of a child, presence of a child under 5, interactions between calendar quarter and ethnicity as well as their main effects, interactions between ethnicity and Cr4 and Cr5, and fixed effects for each combination of state and month starting in April 2020. Cr2, Cr3, Cr4, and Cr5 refer to the second quarter of 2020 through the first quarter of 2021. The data is from IPUMS CPS and covers individuals aged 25-65 over the period between April 2017 and March 2021. Robust standard errors are clustered at the household level, and the estimation uses sampling weights

**Table 5:** The Probability of Transitioning into Employment with Controls for Demographics and Time-varying Labor Market Conditions

	Men HS or less	Men Some Coll.	Men College+	Women HS or less	Women Some Coll.	Women College+
Black*Cr2	−0.018	−0.002	0.020	−0.006	−0.061***	−0.015
	(0.016)	(0.028)	(0.041)	(0.013)	(0.017)	(0.024)
Hispanic*Cr2	0.001	−0.046	0.003	−0.011	−0.007	−0.001
	(0.020)	(0.030)	(0.043)	(0.011)	(0.019)	(0.022)
AsianEast*Cr2	−0.007	−0.020	0.001	−0.004	−0.041	0.024
	(0.035)	(0.047)	(0.043)	(0.023)	(0.030)	(0.022)
AsianOther*Cr2	−0.157***	−0.024	0.028	0.003	0.041	0.021
	(0.048)	(0.062)	(0.046)	(0.025)	(0.043)	(0.021)
Black*Cr3	0.008	−0.007	−0.049	−0.013	0.009	0.033
	(0.017)	(0.028)	(0.036)	(0.014)	(0.019)	(0.025)
Hispanic*Cr3	−0.032	0.039	0.011	−0.001	0.002	0.030
	(0.021)	(0.034)	(0.043)	(0.012)	(0.021)	(0.024)
AsianEast*Cr3	0.019	0.139**	0.018	0.041	0.049	0.002
	(0.042)	(0.059)	(0.042)	(0.027)	(0.038)	(0.024)
AsianOther*Cr3	−0.049	0.027	0.065	0.045	−0.131***	0.019
	(0.052)	(0.061)	(0.058)	(0.036)	(0.042)	(0.024)
Observations	109,304	55,746	49,149	158,109	99,364	106,961

dependent variable is working, and the sample is restricted to those who did not have a job in the previous month. The control variables not reported are: age, age$^2$, marital status, presence of a child, presence of a child under 5, interactions between calendar quarter and ethnicity as well as their main effects, interactions between ethnicity and Cr4 and Cr5, and fixed effects for each combination of state and month starting in April 2020. Cr2, Cr3, Cr4, and Cr5 refer to the second quarter of 2020 through the first quarter of 2021. The data is from IPUMS CPS and covers individuals aged 25-65 over the period between April 2017 and March 2021. Robust standard errors are clustered at the household level, and the estimation uses sampling weights

Table 4 confirms the results in Fig. 2. After controlling for demographics, Asian Americans without a college degree suffered larger job loss in the second quarter of 2020 than other groups. The estimated effects on the probability of entering employment reported in Table 5 tend to be statistically insignificant.

The second motivation for studying transitions is that some industries and occupations were particularly hard hit by the pandemic. This could lead to omitted variable bias if Asian Americans tend to work in those industries or occupations. For example, in the sample used below, East Asians are overrepresented in the occupation “hosts and hostesses, restaurant, lounge, and coffee shop” by a factor of approximately 3 relative to the rest of the population.

The CPS includes information on the industry and occupation to which an individual belongs. In principle, these variables should be well-defined whether or not an individual is currently working as long as they have worked in the past. As such, it should only be missing for individuals who have no attachment to the labor market, and one thus might be able to justify ignoring those individuals from the empirical analysis. However, if—contrary to protocol—the non-response to the questions about industry or occupation is a direct result of not working, then ignoring those individuals will result in selection bias. Indeed, we find strong evidence that non-response to the questions about industry or occupation is caused by not working. For example, consider individuals who worked in the previous month, and hence should in principle report industry and occupation status in the current month regardless of their current employment status. In this group, 31% of those not currently working report occupation as missing, while only 2% of those working report occupation as missing. The corresponding numbers for industry are 30% and 0%, respectively. One potential solution to this problem is to use lagged industry or occupation as an explanatory variable. However, whether this variable is missing might be the consequence of lagged unemployment, which in turn could be highly correlated with current employment status. Again, this could lead to endogeneity in whether or not lagged industry or occupation is missing. In contrast, the transitions in and out of employment are much more likely to be driven by recent events which are less likely to be related to whether or not lagged industry or occupation is missing. Moreover, lagged industry or occupation are rarely missing conditional on the person working in the previous month.^6^

In order to control for occupation-specific impact of the pandemic, we include fixed effects for 5,475 interactions between lagged occupation (including “missing”) and pandemic months. In other words, we modify the model in (1) to

2 $$\begin{aligned} y_{it}&=x_{it}^{\prime }\beta +\lambda _{State_{it},Month_{t}} +\vartheta _{Occupation_{i,t-1}}+\zeta _{CrMonth_{t},Occupation_{it-1}} \nonumber \\&\quad +\gamma _{Ethnicity_{i},Quarter_{t}} +\delta _{Ethnicity_{i},Cr_{t}}+error_{it} \end{aligned}$$

where $Occupation_{i,t-1}$ is the lagged occupation for individual *i* and $CrMonth_{t}$ is a set of dummy variables for each of the crisis months (starting in April 2020). The results are presented in Tables 6 and 7.^7^

**Table 6:** The Probability of Remaining Employed with Controls for Demographics and Time-varying Labor Market Conditions and Time-varying Effects of Occupation

	Men HS or less	Men Some Coll.	Men College+	Women HS or less	Women Some Coll.	Women College+
Black*Cr2	−0.039**	−0.005	−0.035***	−0.047***	−0.005	−0.015
	(0.015)	(0.014)	(0.013)	(0.017)	(0.014)	(0.011)
Hispanic*Cr2	−0.021**	−0.019	−0.006	0.010	−0.014	−0.026**
	(0.010)	(0.013)	(0.010)	(0.016)	(0.015)	(0.012)
AsianEast*Cr2	−0.150***	−0.096***	−0.019	−0.093**	−0.063*	−0.019
	(0.038)	(0.033)	(0.012)	(0.038)	(0.032)	(0.012)
AsianOther*Cr2	−0.073**	−0.101***	−0.014*	−0.026	−0.068	0.011
	(0.037)	(0.038)	(0.009)	(0.040)	(0.043)	(0.012)
Black*Cr3	−0.005	−0.011	0.000	−0.024	−0.042***	−0.010
	(0.012)	(0.013)	(0.011)	(0.015)	(0.014)	(0.010)
Hispanic*Cr3	−0.009	0.001	−0.001	0.011	−0.001	−0.017
	(0.008)	(0.010)	(0.010)	(0.013)	(0.012)	(0.012)
AsianEast*Cr3	−0.010	−0.039	−0.008	−0.049*	−0.014	−0.012
	(0.020)	(0.024)	(0.010)	(0.026)	(0.021)	(0.011)
AsianOther*Cr3	0.003	−0.012	0.008	0.046	0.079***	−0.002
	(0.026)	(0.030)	(0.008)	(0.039)	(0.023)	(0.015)
Observations	268,219	195,225	295,552	180,794	193,398	311,497

The dependent variable is working, and the sample is restricted to those who had a job in the previous month. The control variables not reported are: age, age$^2$, marital status, presence of a child, presence of a child under 5, interactions between calendar quarter and ethnicity as well as their main effects, fixed effects for each combination of state and month starting in April 2020, interactions between ethnicity and Cr4 and Cr5, and fixed effects for lagged occupation (using the 2010 Census occupation coding scheme) and each combination of lagged occupation and month starting in April 2020. Cr2, Cr3, Cr4, and Cr5 refer to the second quarter of 2020 through the first quarter of 2021. The data is from IPUMS CPS and covers individuals aged 25-65 over the period between April 2017 and March 2021. Robust standard errors are clustered at the household level, and the estimation uses sampling weights

**Table 7:** The Probability of Transitioning into Employment with Controls for Demographics and Time-varying Labor Market Conditions and Time-varying Effects of Occupation

	Men HS or less	Men Some Coll.	Men College+	Women HS or less	Women Some Coll.	Women College+
Black*Cr2	−0.003	0.022	0.009	−0.002	−0.039***	0.004
	(0.015)	(0.024)	(0.034)	(0.012)	(0.015)	(0.020)
Hispanic*Cr2	−0.025	−0.039	0.008	−0.019**	0.005	−0.015
	(0.019)	(0.027)	(0.042)	(0.009)	(0.017)	(0.019)
AsianEast*Cr2	−0.051	0.007	0.022	−0.045**	−0.053*	0.012
	(0.036)	(0.044)	(0.040)	(0.021)	(0.027)	(0.018)
AsianOther*Cr2	−0.143***	0.020	0.030	−0.009	−0.006	0.019
	(0.045)	(0.065)	(0.042)	(0.024)	(0.032)	(0.018)
Black*Cr3	0.008	−0.002	−0.058*	−0.013	0.008	0.036*
	(0.015)	(0.025)	(0.032)	(0.012)	(0.017)	(0.021)
Hispanic*Cr3	−0.044**	0.033	0.062	-0.015	−0.007	0.010
	(0.020)	(0.032)	(0.040)	(0.011)	(0.017)	(0.021)
AsianEast*Cr3	0.010	0.117**	0.038	−0.014	0.032	−0.007
	(0.039)	(0.054)	(0.040)	(0.023)	(0.037)	(0.021)
AsianOther*Cr3	−0.055	0.040	0.077	0.029	−0.066	−0.015
	(0.051)	(0.055)	(0.053)	(0.031)	(0.043)	(0.020)
Observations	109,304	55,746	49,149	158,109	99,364	106,961

The dependent variable is working, and the sample is restricted to those who did not have a job in the previous month. The control variables not reported are: age, age$^2$, marital status, presence of a child, presence of a child under 5, interactions between calendar quarter and ethnicity as well as their main effects, interactions between ethnicity and Cr4 and Cr5, fixed effects for each combination of state and month starting in April 2020, and fixed effects for lagged occupation (using the 2010 Census occupation coding scheme) and each combination of lagged occupation and month starting in April 2020. Cr2, Cr3, Cr4, and Cr5 refer to the second quarter of 2020 through the first quarter of 2021. The data is from IPUMS CPS and covers individuals aged 25-65 over the period between April 2017 and March 2021. Robust standard errors are clustered at the household level, and the estimation uses sampling weights

A comparison of the results in Tables 4 and 6 reveals that an individual’s occupation can explain up to approximately half of the difference between Whites and the two groups of Asian Americans. For example, for East and South-East Asian American men with a high school degree or less, the estimated effect on the probability of remaining employed (relative to comparable Whites) is reduced from $-0.22$ to $-0.15$ when one controls for time-varying occupation effects. The corresponding reduction for women is from $-0.20$ to $-0.09$.

The same comparison for the probability of entering employment (Tables 5 and 7) suggests that controlling for occupation makes only a small difference relative to the estimation uncertainty.

## Other possible explanations

### Anti-Asian sentiment

It is possible that the experience of Asian American workers during the pandemic reflects the impact of increased discrimination due to their perceived association with China. Such an explanation would be consistent with Kaushal et al. (2007). That paper finds that earnings of Arab and Muslim men declined dramatically after the September 11th terrors’ attack. In line with this, Shin (2021) finds a negative employment effect of the attack for Arab and African refugees. It would also be consistent with the finding in Sakong (2018) that economic downturns are often associated with increased racial prejudice.

We take two approaches to investigating this. The first approach is to combine the CPS data with a state specific measure of anti-Asians bias, and investigate whether Asian Americans in states with larger bias were harder hit by the pandemic than those in states with less bias. The second approach is to investigate whether Asian American working in jobs that involve more intensive interpersonal interactions are harder hit than those in other occupations.

We construct a measure of racial bias using data from the Project Implicit Database.^8^ Specifically, we use data from respondents of the “Asian Implicit Association Test” from 2004 to 2020 and construct a variable “implicit bias” as the average IAT score by month and state (see, e.g. Darling-Hammond et al. (2020)). We then augment the specifications in Table 6 by adding interactions between this bias variable and the pandemic-Asian interactions. In order to avoid potential reverse causality, we use the average values of the implicit bias in a state in 2018. When doing this, we find no evidence that Asian Americans in states that had stronger anti-Asian bias in 2018 saw an especially large decline in employment during the pandemic.

Our second approach for detecting Anti-Asian sentiment is to look at whether Asian Americans are especially negatively impacted in occupations that involve intensive interpersonal interactions. To do this, we combine the CPS data with the mapping from occupation to tasks developed by Aaronson and Phelan (2020).^9^ Specifically, we use whether the variable “interpersonal task share” exceeds 0.5 (corresponding to the 75*th* percentile). This includes, for example, the occupations “sales representatives, wholesale and manufacturing”, “bartender”, “host and hostesses, restaurant, lounge and coffee shops”, and “real estate brokers and sales agents”. On the other hand, the lower tail of “interpersonal task share” includes, for example, the occupations “industrial truck and tractor operators”, “welding, soldering and brazing workers” and “electrical, electronic, electromechanical assemblers”. The variable, interpersonal task share (“IP” in the table), is interacted with the indicator variables for being Asian American, as well as their interactions with the pandemic quarters.^10^ These are used as additional explanatory variables in the regression in Table 6. The estimates are reported in Table 8. Only two of the estimated coefficients in Table 8 are negative and statistically significant at the 5 percent level. With 24 estimated parameters, this is not very different from what one might expect if all the parameter values are truly 0.

**Table 8:** The Disparate Effect of Job Characteristics on The Probability of Remaining Employed

	Men HS or less	Men Some Coll.	Men College+	Women HS or less	Women Some Coll.	Women College+
AsianEast*IP*Cr2	−0.018	−0.130**	−0.038*	0.026	0.094*	0.013
	(0.084)	(0.066)	(0.021)	(0.073)	(0.057)	(0.021)
AsianOther*IP*Cr2	−0.050	−0.009	0.017	−0.013	−0.123	−0.046**
	(0.067)	(0.076)	(0.017)	(0.070)	(0.083)	(0.019)
AsianEast*IP*Cr3	−0.056	−0.089	0.026	−0.060	0.026	−0.020
	(0.077)	(0.062)	(0.017)	(0.060)	(0.036)	(0.017)
AsianOther*IP*Cr3	−0.097	−0.055	0.029**	0.053	−0.029	−0.014
	(0.076)	(0.069)	(0.014)	(0.063)	(0.031)	(0.027)
Observations	268,219	195,225	295,552	180,794	193,398	311,497

The dependent variable is working, and the sample is restricted to those who had a job in the previous month. The control variables not reported are: age, age$^2$, marital status, presence of a child, presence of a child under 5, interactions between calendar quarter and ethnicity as well as their main effects, interactions between ethnicity and Cr2 through Cr5, fixed effects for each combination of state and month starting in April 2020, interactions between Asians, CR4 and Cr5 and occupation-specific intensity of interpersonal tasks (IP), and fixed effects for lagged occupation (using the 2010 Census occupation coding scheme) and each combination of lagged occupation and month starting in April 2020. Cr2, Cr3, Cr4, and Cr5 refer to the second quarter of 2020 through the first quarter of 2021. The data is from IPUMS CPS and covers individuals aged 25-65 over the period between April 2017 and March 2021. The variable IP is a dummy variable for whether “interpersonal task share” for the occupation exceeds 0.5 (which corresponds to the 75th percentile). The mapping from occupation to “interpersonal task share” is based on O*NET (see Acemoglu and Autor (2011) and Aaronson and Phelan (2020)). Robust standard errors are clustered at the household level, and the estimation uses sampling weights

### Household composition

Another potential explanation for the differential downturn in employment across ethnicities is that some groups are more likely to live in multi-generational households or more likely to have small children. Both of these could reduce labor supply in a way that differs across gender, ethnicity, and education groups.

If people are concerned about the health of the older members and stop working during the pandemic as a result of this, then this might induce different patterns in employment across ethnicities. The results in Table 9 suggest that this is not the explanation for our findings. When we restrict the sample to individuals in households where there is no member older than 65 years, the estimates are very close to those in Table 6.

**Table 9:** The Probability of Remaining Employed with Controls for Demographics and Time-varying Labor Market Conditions and Time-varying Effects of Occupation, Excluding Individuals With Household Members Older Than 65

	Men HS or less	Men Some Coll.	Men College+	Women HS or less	Women Some Coll.	Women College+
Black*Cr2	−0.045***	0.001	−0.038***	−0.055***	−0.001	−0.017
	(0.016)	(0.014)	(0.014)	(0.018)	(0.014)	(0.012)
Hispanic*Cr2	−0.018	−0.014	−0.010	0.004	−0.007	−0.023*
	(0.011)	(0.013)	(0.010)	(0.016)	(0.015)	(0.013)
AsianEast*Cr2	−0.132***	−0.088**	−0.017	−0.089**	−0.063*	−0.014
	(0.041)	(0.034)	(0.012)	(0.040)	(0.035)	(0.013)
AsianOther*Cr2	−0.064	−0.094**	−0.011	−0.024	−0.095*	0.012
	(0.041)	(0.041)	(0.008)	(0.048)	(0.053)	(0.012)
Black*Cr3	−0.002	−0.012	−0.006	−0.031*	−0.039***	−0.013
	(0.012)	(0.014)	(0.011)	(0.016)	(0.015)	(0.010)
Hispanic*Cr3	−0.007	−0.000	−0.002	0.010	0.003	−0.011
	(0.009)	(0.010)	(0.009)	(0.014)	(0.012)	(0.012)
AsianEast*Cr3	−0.007	−0.044	−0.014	−0.063**	−0.014	−0.015
	(0.021)	(0.027)	(0.010)	(0.030)	(0.024)	(0.012)
AsianOther*Cr3	0.003	0.023	0.010	0.029	0.069***	−0.004
	(0.027)	(0.024)	(0.008)	(0.046)	(0.023)	(0.016)
Observations	245,295	181,385	280,638	160,000	174,935	287,249

The dependent variable is working, and the sample is restricted to those who had a job in the previous month and who did not have a household member over the age of 65. The control variables not reported are: age, age$^2$, marital status, presence of a child, presence of a child under 5, interactions between calendar quarter and ethnicity as well as their main effects, interactions between ethnicity and Cr4 and Cr5, fixed effects for each combination of state and month starting in April 2020, and fixed effects for lagged occupation (using the 2010 Census occupation coding scheme) and each combination of lagged occupation and month starting in April 2020. Cr2, Cr3, Cr4, and Cr5 refer to the second quarter of 2020 through the first quarter of 2021. The data is from IPUMS CPS and covers individuals aged 25-65 over the period between April 2017 and March 2021. Robust standard errors are clustered at the household level, and the estimation uses sampling weights

The presence of a young child in a household could increase parents’ reservation wages during the pandemic period. For example, the shortage of child care could make it more likely for mothers to stop working in a way that differs across ethnicity and education groups. To investigate this, we augmented the model in Table 6 by adding interactions between the presence of a child under the age of five, the pandemic quarters and ethnicity. The p-values for the joint test that the coefficients on these are all 0 are presented in Table 10 for the first and the second quarter of the pandemic. None of the p-values is below 0.05 and their average is only slightly below one half. This suggests that the presence of small children in the household does not have an effect that differs across ethnicities in an important way.

**Table 10:** Joint Testing for the Disparate Employment-Effect of Young Children Across Ethnicities

	Men HS or less	Men Some Coll.	Men College+	Women HS or less	Women Some Coll.	Women College+
Cr2 * Ethnicity * Child under 5	0.6208	0.1499	0.4875	0.0953	0.6568	0.0778
Cr3 * Ethnicity * Child under 5	0.2043	0.0751	0.0670	0.6950	0.4012	0.0708

The dependent variable is working, and the sample is restricted to those who had a job in the previous month. The numbers reported are the p-values for the joint test that the coefficients on the interactions between the crisis quarters, ethnicity and the presence a child under 5 are all zero. The control variables not reported are: age, age$^2$, marital status, presence of a child, presence of a child under 5, interactions between calendar quarter and ethnicity as well as their main effects, interactions between ethnicity and Cr2 through Cr5, interactions between ethnicity, Cr4 and Cr5, and presence of a child under 5, and fixed effects for each combination of state and month starting in April 2020. Cr2, Cr3, Cr4, and Cr5 refer to the second quarter of 2020 through the first quarter of 2021. The data is from IPUMS CPS and covers individuals aged 25–65 over the period between April 2017 and March 2021. Robust standard errors are clustered at the household level, and the estimation uses sampling weights

Overall, the results regarding multi-generational households and regarding the presence of small children in the household suggest that the large effect of the pandemic on the employment of low-educated Asian Americans is not primarily driven by supply-side effects.

### Country of birth

One distinct feature of Asian Americans is that they are more likely to be foreign born than the other ethnicities. For example, $21\%$ of the total sample is foreign born, while the fraction for Asian Americans is $73\%$. As a comparison, the numbers are 6%, 17%, and 57% for Whites, Blacks and Hispanics, respectively. This suggests that the labor market differences between Asian Americans and Whites could be associated with language obstacles, other cultural differences associated with being foreign born, or discrimination against foreigners. In order to test this, we re-estimate the main model in Table 6 separately for the sample of individuals who are born in the United States and for sample of those who are not.

The results for the US born are presented in Table 11. It is striking that all of the estimates related to Asian Americans interacted with the second quarter of 2020 are now statistically insignificant at the $5\%$ level.

**Table 11:** The Probability of Remaining Employed with Controls for Demographics and Time-varying Labor Market Conditions and Time-varying Effects of Occupation, U.S. Born Only

	Men HS or less	Men Some Coll.	Men College+	Women HS or less	Women Some Coll.	Women College+
Black*Cr2	−0.023	−0.003	−0.034**	−0.060***	−0.007	−0.007
	(0.016)	(0.015)	(0.016)	(0.019)	(0.014)	(0.012)
Hispanic*Cr2	0.003	−0.013	0.022*	0.011	−0.027	−0.009
	(0.015)	(0.016)	(0.012)	(0.021)	(0.018)	(0.015)
AsianEast*Cr2	−0.077	−0.029	0.010	0.093	0.010	−0.011
	(0.072)	(0.048)	(0.019)	(0.083)	(0.054)	(0.023)
AsianOther*Cr2	−0.068	−0.075*	−0.020	−0.122	−0.053	0.007
	(0.058)	(0.044)	(0.020)	(0.091)	(0.061)	(0.020)
Black*Cr3	−0.010	−0.004	0.004	−0.024	−0.047***	−0.010
	(0.014)	(0.014)	(0.013)	(0.017)	(0.015)	(0.011)
Hispanic*Cr3	−0.017	0.001	−0.003	−0.010	−0.002	−0.027*
	(0.014)	(0.012)	(0.013)	(0.018)	(0.014)	(0.015)
AsianEast*Cr3	0.021	0.027	−0.001	−0.090	−0.098	0.002
	(0.031)	(0.031)	(0.016)	(0.065)	(0.065)	(0.016)
AsianOther*Cr3	−0.052	−0.074	0.026*	0.077	0.082***	−0.011
	(0.061)	(0.055)	(0.014)	(0.068)	(0.030)	(0.029)
Observations	205,435	174,011	244,539	139,313	173,056	266,264

The dependent variable is working, and the sample is restricted to those who had a job in the previous month and who were born in the U.S.A. The control variables not reported are: age, age$^2$, marital status, presence of a child, presence of a child under 5, interactions between calendar quarter and ethnicity as well as their main effects, interactions between ethnicity and Cr4 and Cr5, fixed effects for each combination of state and month starting in April 2020, and fixed effects for lagged occupation (using the 2010 Census occupation coding scheme) and each combination of lagged occupation and month starting in April 2020. Cr2, Cr3, Cr4, and Cr5 refer to the second quarter of 2020 through the first quarter of 2021. The data is from IPUMS CPS and covers individuals aged 25-65 over the period between April 2017 and March 2021. Robust standard errors are clustered at the household level, and the estimation uses sampling weights

Table 12 presents the results for non-U.S. born individuals. The estimation uncertainty is much greater in this sample, but—as one might expect—the point estimates of the impact of the pandemic on Asian Americans are larger for this subgroup than for the corresponding sample that includes US-born individuals. We conclude that the group that stands out is foreign born, low educated Asian Americans. We have no direct evidence about the reason, but one might speculate that this is due to cultural differences or language barriers.

**Table 12:** The Probability of Remaining Employed with Controls for Demographics and Time-varying Labor Market Conditions and Time-varying Effects of Occupation, Not U.S. Born

	Men HS or less	Men Some Coll.	Men College+	Women HS or less	Women Some Coll.	Women College+
Black*Cr2	−0.108**	−0.027	−0.037	−0.016	0.014	−0.028
	(0.047)	(0.069)	(0.024)	(0.058)	(0.070)	(0.034)
Hispanic*Cr2	−0.053*	0.048	−0.045**	0.066	0.062	−0.055*
	(0.028)	(0.053)	(0.022)	(0.049)	(0.054)	(0.030)
AsianEast*Cr2	−0.185***	−0.070	−0.021	−0.114	−0.006	−0.004
	(0.054)	(0.070)	(0.018)	(0.070)	(0.063)	(0.023)
AsianOther*Cr2	−0.082	−0.263***	−0.010	0.004	−0.090	0.022
	(0.055)	(0.092)	(0.014)	(0.075)	(0.094)	(0.024)
Black*Cr3	0.011	−0.054	0.011	−0.000	0.003	0.001
	(0.032)	(0.048)	(0.024)	(0.042)	(0.044)	(0.031)
Hispanic*Cr3	0.019	−0.020	0.008	0.031	0.035	0.003
	(0.022)	(0.028)	(0.018)	(0.036)	(0.041)	(0.023)
AsianEast*Cr3	0.021	−0.070	−0.008	−0.000	0.059	−0.009
	(0.033)	(0.044)	(0.016)	(0.046)	(0.048)	(0.019)
AsianOther*Cr3	0.030	0.013	−0.006	0.066	0.106*	0.008
	(0.039)	(0.037)	(0.015)	(0.056)	(0.058)	(0.022)
Observations	62,784	21,214	51,013	41,481	20,342	45,233

The dependent variable is working, and the sample is restricted to those who had a job in the previous month and were not born in the U.S.A. The control variables not reported are: age, age$^2$, marital status, presence of a child, presence of a child under 5, interactions between calendar quarter and ethnicity as well as their main effects, interactions between ethnicity and Cr4 and Cr5, fixed effects for each combination of state and month starting in April 2020, and fixed effects for lagged occupation (using the 2010 Census occupation coding scheme) and each combination of lagged occupation and month starting in April 2020. Cr2, Cr3, Cr4, and Cr5 refer to the second quarter of 2020 through the first quarter of 2021. The data is from IPUMS CPS and covers individuals aged 25–65 over the period between April 2017 and March 2021. Robust standard errors are clustered at the household level, and the estimation uses sampling weights

### Summary

At the suggestion of an anonymous referee, we quantify the relative importance of the aforementioned explanations by a Blinder-Oaxaca-type decomposition composed of three steps. First, for each variable, we “partial out” the effects of the demographic controls (age, age$^2$, marital status, presence of a child, presence of a child under 5), interactions between calendar quarter and ethnicity as well as their main effects, fixed effects for each combination of state and month starting in April 2020, and fixed effects for lagged occupation and each combination of lagged occupation and month starting in April 2020. Secondly, for each ethnicity, we regress the residualized dependent variable (working) on the measurement of the five explanations of interest (anti-Asian sentiment, characteristics of the job, presence of small children, presence of elderly household members, and country of birth) interacted with the crisis quarters. Finally, we calculate the mean counterfactual outcomes that result from combining variables for one ethnicity group with parameter estimates from another group.

Table 13 reports the point estimates for the decomposition of the effect of Cr2 for the subsample with a high school degree or less. Specifically, for each minority group, the first column displays the difference between “*x*-beta” for Whites and the group , $\bar{x}_W\beta _W - \bar{x}_g\beta _g$ (the “total” gap). The second and third column decompose these into the average characteristic for Whites multiplied by the difference in the estimate, $\bar{x}_W(\beta _W - \beta _g$) (the “structural effect”), and the difference in the average characteristics multiplied by the estimates for the group, $(\bar{x}_W- \bar{x}_g)\beta _g$ (the “endowment effect”), respectively.

The most consistent result in Table 13 is that there is very little difference in the effect of household composition (Kids and Elderly) across groups. This conclusion remains when we combine characteristics of one group with parameter estimates for another group. The results for the other variables are more mixed. For both men and women, the largest numbers are those for being born in the United States. These numbers are especially large for Asian East, and they are almost entirely driven by the endowment effect, i.e., the difference between Whites and the other groups in the probability of being US-born. The only other relatively large difference between the effects for Whites and for Asia East is in the effect of anti-Asian sentiment for men. As one might expect, the decomposition suggests that this difference is driven by the difference in the parameter estimates as opposed to geographic exposure to the sentiment.

**Table 13:** Blinder–Oaxaca-type Decomposition

	Blacks			Hispanics			Asians East			Asians Other		
	Tot.	Str.	End.	Tot.	Str.	End.	Tot.	Str.	End.	Tot.	Str.	End.
*Men*												
Bias	3.57	3.75	$-$ 0.17	0.54	1.03	$-$ 0.49	$-$ 5.20	$-$ 7.55	2.35	$-$ 1.03	$-$ 0.95	$-$ 0.08
Tasks	2.57	1.11	1.45	0.59	2.51	$-$ 1.92	1.69	1.29	0.41	3.38	2.93	0.45
USborn	0.64	0.13	0.51	1.89	0.04	1.85	6.50	0.24	6.26	0.46	$-$ 0.03	0.50
Kids	0.55	0.57	$-$ 0.02	$-$ 0.02	$-$ 0.01	$-$ 0.01	0.92	0.89	0.02	$-$ 0.03	$-$ 0.02	$-$ 0.01
Elderly	$-$ 0.15	$-$ 0.18	0.03	0.11	0.24	$-$ 0.13	0.29	0.08	0.22	1.03	0.41	0.61
*Women*												
Bias	2.95	3.24	$-$ 0.29	$-$ 2.14	$-$ 2.23	0.09	$-$ 0.27	0.52	$-$ 0.79	$-$ 4.39	$-$ 6.10	1.71
Tasks	0.03	$-$ 0.32	0.35	6.04	6.37	$-$ 0.33	2.20	3.45	$-$ 1.25	1.18	0.71	0.47
USborn	$-$ 0.66	$-$ 0.43	$-$ 0.23	1.14	$-$ 0.24	1.38	17.58	0.58	17.00	$-$ 5.59	$-$ 0.72	$-$ 4.88
Kids	$-$ 0.18	$-$ 0.19	0.01	$-$ 1.16	$-$ 0.86	$-$ 0.30	0.28	0.35	$-$ 0.07	1.21	0.61	0.61
Elderly	$-$ 0.62	$-$ 0.58	$-$ 0.04	0.09	0.65	$-$ 0.56	0.49	0.11	0.39	0.66	0.22	0.44

For each minority group, the first column displays the difference between “*x*-beta” for whites and the group, $\bar{x}_W\hat{\beta }_W - \bar{x}_g\hat{\beta }_g$ (i.e., the total gap). The second and third columns decompose these into the average characteristic for Whites multiplied by the difference in the estimate, $\bar{x}_W(\hat{\beta }_W - \hat{\beta }_g )$ (i.e., the structural effect), and the difference in the average characteristics multiplied by the estimates for the group, $(\bar{x}_W-\bar{x}_g)\hat{\beta }_g$ (i. e., the endowment effect), respectively. The results are based on the three-step procedure outlined in the text. The numbers are scaled by 100 in order to make the effect in percentage points

## The role of education

The analysis so far has been done separately for different education groups. One could argue that education is a choice made by an individual, and that this would make it endogenous. Table 14 shows the distribution of education by ethnicity for both genders in our sample. It is very clear that Asian Americans have higher education on average than other groups. In other words, the selection into education level potentially differs across the ethnicities. For example, the group of Asians Americans with a high school degree or less might be very different in terms of unobservables from other groups with the same level of education.

**Table 14:** Distribution of Education (Aged 25–65)

	Men HS or less	Men College$+$	Men Some Col.	Women HS or less	Women Some Col.	Women College$+$
Whites	0.338	0.265	0.397	0.272	0.282	0.446
Blacks	0.462	0.288	0.250	0.380	0.318	0.301
Hispanic	0.612	0.212	0.176	0.543	0.238	0.218
Asians	0.232	0.164	0.604	0.244	0.164	0.592
Overall	0.394	0.253	0.353	0.330	0.273	0.397

The data is from IPUMS CPS and covers individuals aged 25–65 over the period between April 2017 and March 2021. The averages are calculated using sampling weights

To investigate whether the results for ethnicity are biased by selection into different education groups, we estimate a model for the probability of working with the same explanatory variables as in Sect. 3, but now using the whole sample without conditioning on education groups. The results in Table 15 show that Blacks, Hispanics and Asians all experienced a bigger impact of the crisis on their employment than Whites. The point estimates are especially large for East and South-East Asians who had the largest initial drops in employment for both men and women.^11^ This is consistent with the findings in Sect. 3.

**Table 15:** The Probability of Working with Controls for Demographics and Time-varying Labor Market Conditions, Not Conditional on Education

	Men	Women
Black*Cr2	−0.050***	−0.031***
	(0.011)	(0.010)
Hispanic*Cr2	−0.057***	−0.035***
	(0.009)	(0.010)
AsianEast*Cr2	−0.072***	−0.062***
	(0.015)	(0.016)
AsianOther*Cr2	−0.033**	0.051***
	(0.015)	(0.019)
Black*Cr3	–0.041***	−0.042***
	(0.010)	(0.009)
Hispanic*Cr3	−0.030***	−0.013
	(0.008)	(0.009)
AsianEast*Cr3	−0.008	−0.015
	(0.015)	(0.015)
AsianOther*Cr3	0.010	0.012
	(0.014)	(0.019)
Observations	1,395,466	1,506,023

The dependent variable is working. The control variables not reported are: age, age$^2$, marital status, presence of a child, presence of a child under 5, interactions between calendar quarter and ethnicity as well as their main effects, interactions between ethnicity and Cr4 and Cr5, and fixed effects for each combination of state and month starting in April 2020. Cr2, Cr3, Cr4, and Cr5 refer to the second quarter of 2020 through the first quarter of 2021. The data is from IPUMS CPS and covers individuals aged 25-65 over the period between April 2017 and March 2021. Robust standard errors are clustered at the household level, and the estimation uses sampling weights

## Conclusion

This paper has documented that Asian Americans with no college education were especially hard hit by the onset of the pandemic. The negative employment effect on Asian Americans with no college education remains after controlling for differences in demographics, local labor market conditions, and job characteristics. The extra burden is primarily borne by individuals who were born outside the United States. The results add to the growing evidence that the pandemic has had very different effects across different ethnicities. Here, we have studied employment. Whether the results generalize to other economic outcomes is an interesting topic for future research.

The paper illustrates the importance of treating Asian Americans as a distinct minority. Highly educated Asian Americans are similar to Whites in terms of labor market outcomes, but lower educated Asian Americans are more similar to other disadvantaged minority groups. This also highlights the vast heterogeneity within Asian Americans (Kochhar and Cilluffo 2018). Since Asian Americans is the fastest growing ethnic group in the United States (see Budiman and Ruiz (2021)), we expect these considerations to be even more important in future research.

## Supplementary Information

Below is the link to the electronic supplementary material.

Supplementary file 1 (pdf 197 KB)

## Footnotes

1 See the tables in the Supplementary Material.

2 These are self-reported, and hence subject to possible misclassification.

3 Additional details are presented in the Supplementary Material Tables 1 and 2 in the Supplemental Material.

4 Similar patterns hold if one changes the definition of working to also include those who report “has a job, but not at work last week”. See Honoré and Hu (2021). We do not pursue this further because this alternative definition of working has been subject to a great deal of mis-classification since the beginning of the pandemic (See US Bureau of Labor Statistics (2020)).

5 For comparison, the probability of working in the quarter prior to the onset of the pandemic was 72% and 54% for two groups.

6 Indeed, in the estimation we did not lose any observations because of this.

7 We repeated the same exercise using industry rather than occupation. The results are very similar, although it appears that controlling for occupation makes the bigger difference. We refrain from using all combinations of industry, occupation, and month because this would lead to many “cells” with only one observation, and the fixed effects approach would effectively drop these observations from the sample.

8 https://implicit.harvard.edu/implicit/

9 Aaronson and Phelan (2020) in turn builds on Acemoglu and Autor (2011), who developed the measures from the O*NET database.

10 Since the other ethnicity groups did not experience as dramatic a drop in employment at the onset of the pandemic, we did not attempt to decompose their decline into whether or not they were in an occupation with high level of interpersonal interactions.

11 Interestingly, for men, the estimated effects for Cr2 are not statistically significantly different between minority groups. For women, the effect for AsianOther is different from any other group. For both genders, the effect for any minority group is different from the effect for Whites.
