# Supplementary material for: The COVID-19 pandemic and Asian American employment
Source: Empir Econ. 2022 Oct 29;64(5):2053–83. doi: 10.1007/s00181-022-02306-5 (PMC9617602; doi:10.1007/s00181-022-02306-5)
Supplement: Supplementary file 1 — (pdf 197 KB) [file 181_2022_2306_MOESM1_ESM.pdf]

Supplementary Material

1 Additional Summary Statistics

This section presents two tables of additional summary statistics, which originally appeared in the unpublished version of the paper.

Supplementary Material Table 1: Fraction of Men Working by Group in 2020-21

	Period	HS or less	Some Col.	College+	Overall
Whites	Jan-Mar, 2020	0.708	0.789	0.873	0.795
	Apr-Jun	0.621	0.699	0.819	0.723
	Jul-Sep	0.681	0.740	0.821	0.754
	Oct-Dec	0.684	0.742	0.857	0.769
	Jan-Mar, 2021	0.657	0.748	0.861	0.764
Blacks	Jan-Mar, 2020	0.578	0.735	0.858	0.697
	Apr-Jun	0.482	0.636	0.731	0.589
	Jul-Sep	0.543	0.666	0.757	0.631
	Oct-Dec	0.558	0.686	0.774	0.651
	Jan-Mar, 2021	0.545	0.676	0.804	0.651
Hispanics	Jan-Mar, 2020	0.800	0.828	0.893	0.823
	Apr-Jun	0.679	0.692	0.782	0.702
	Jul-Sep	0.736	0.766	0.796	0.755
	Oct-Dec	0.764	0.794	0.824	0.781
	Jan-Mar, 2021	0.752	0.746	0.850	0.769
Asians	Jan-Mar, 2020	0.768	0.763	0.871	0.832
	Apr-Jun	0.459	0.559	0.799	0.692
	Jul-Sep	0.631	0.712	0.838	0.774
	Oct-Dec	0.684	0.754	0.865	0.805
	Jan-Mar, 2021	0.681	0.722	0.864	0.802
All	Jan-Mar, 2020	0.715	0.786	0.873	0.790
	Apr-Jun	0.608	0.682	0.805	0.700
	Jul-Sep	0.671	0.732	0.815	0.740
	Oct-Dec	0.686	0.742	0.847	0.758
	Jan-Mar, 2021	0.666	0.736	0.855	0.753

The underlying data is from IPUMS CPS and covers individuals aged 25-65 over the period between April 2017 and March 2021. The averages are calculated using sampling weights.

Supplementary Material Table 2: Fraction of Women Working by Group in 2020-21

	Period	HS or less	Some Col.	College+	Overall
Whites	Jan-Mar, 2020	0.558	0.673	0.779	0.690
	Apr-Jun	0.445	0.571	0.687	0.593
	Jul-Sep	0.502	0.611	0.689	0.620
	Oct-Dec	0.527	0.649	0.757	0.667
	Jan-Mar, 2021	0.519	0.652	0.762	0.670
Blacks	Jan-Mar, 2020	0.558	0.679	0.784	0.669
	Apr-Jun	0.422	0.540	0.715	0.554
	Jul-Sep	0.450	0.588	0.736	0.585
	Oct-Dec	0.495	0.635	0.771	0.627
	Jan-Mar, 2021	0.486	0.628	0.770	0.625
Hispanics	Jan-Mar, 2020	0.526	0.698	0.765	0.619
	Apr-Jun	0.387	0.563	0.626	0.488
	Jul-Sep	0.465	0.587	0.668	0.547
	Oct-Dec	0.488	0.630	0.713	0.574
	Jan-Mar, 2021	0.472	0.629	0.724	0.565
Asians	Jan-Mar, 2020	0.556	0.647	0.671	0.641
	Apr-Jun	0.324	0.448	0.631	0.537
	Jul-Sep	0.459	0.570	0.635	0.587
	Oct-Dec	0.505	0.607	0.657	0.613
	Jan-Mar, 2021	0.521	0.604	0.687	0.635
All	Jan-Mar, 2020	0.549	0.676	0.765	0.672
	Apr-Jun	0.419	0.559	0.678	0.566
	Jul-Sep	0.481	0.601	0.686	0.600
	Oct-Dec	0.510	0.642	0.743	0.642
	Jan-Mar, 2021	0.501	0.642	0.751	0.644

The table shows the fraction of individuals in each group that report to be “at work” in a given month. The underlying data is from IDEMS CPS and covers individuals aged 25-65 over the period between April 2017 and March 2021. The averages are calculated using sampling weights.

2 Robustness

This section contains some robustness checks that appeared in the unpublished version of the paper.

The results presented in the published version are based on estimation of linear probability models. Below, we re-estimate one of the models using a fixed effects logit approach. The results suggest that our conclusions are not driven by this linearity assumption.

We also compare the effects of the pandemic on different ethnic groups to the effects of the Great Recession. We find that in contrast to the pandemic, there was no notable difference in the effects of the 2008 economic downturn on Asians-Americans and Whites. This is perhaps not surprising since the COVID-19 pandemic affected very different sectors of the economy than previous downturns.

2.1 Logit or Linear Probability Model?

The outcome "working" is binary. This leads to the question of whether the fixed effects linear probability model will adequately capture its relationship with the explanatory variables. We therefore investigate whether our results above are likely to be biased by using a linear probability model. This is less straightforward than it might seem because all the models above include interactions which can be thought of as group-specific fixed effects. For example, the estimation leading to Tables 2 to 5 in the paper absorbs 663 interactions between state and month, while the regressions reported in the later tables have up to 5,475 occupation-month interactions. Including this many parameters in a nonlinear model can not only be computationally burdensome, but can also distort the statistical properties of the estimators of all the parameters. This is known as the incidental parameters problem.[1] The traditional solution to this well-known problem is to estimate the parameters (other than the fixed effects) by conditional maximum likelihood. This idea dates back to Rasch (1960, 1961). Unfortunately, this conditional likelihood approach to eliminating the fixed effects is computationally infeasible in our application. We therefore use a modified version of the conditional likelihood approach to check whether our results are sensitive to the use of the linear probability model.

Our approach is based on the same insight that leads to the conditional likelihood estimator mentioned above. Specifically, let i index a group of observations (corresponding to a specific value of the interaction in question), and let j denote an observation within group i. We assume that the dependent variable, y_{ij} (working), for observation j in group i is independent conditional on the explanatory variables, x_{ij}, and on a group-specific effect, α_i. We also assume that the distribution of each y_{ij} is

$$P\left(y_{ij}=1 \middle| \left\{x_{ij}\right\}_{j=1}^{J_i}, \alpha_i\right) = \frac{\exp\left(x_{ij}'\beta+\alpha_i\right)}{1+\exp\left(x_{ij}'\beta+\alpha_i\right)} = \Lambda\left(x_{ij}'\beta+\alpha_i\right), \tag{1}$$

where $\Lambda(\cdot)$ denotes the logistic cumulative distribution function and J_i denotes the number of observations in group i.

[1] See, for example, Neyman and Scott (1948) or Lancaster (2000) for a discussion of the incidental parameters problem. Arellano and Hahn (2007) discuss how incident parameter can be a problem even when the number of observations per group is large, but small relative to the sample size.

In this case, the distribution of $(y_{i1}, \ldots, y_{iJ_i})$ conditional on $(x_{i1}, \ldots, x_{iJ_i})$ and on $\sum_{j=1}^{J_i} y_{ij}$ is

$$P\left(\{y_{ij}\}_{j=1}^{J_i} = \{c_{ij}\}_{j=1}^{J_i} \middle| \{x_{ij}\}_{j=1}^{J_i}, \sum_{j=1}^{J_i} y_{ij} = \sum_{j=1}^{J_i} c_{ij}\right) = \frac{\exp\left(\sum_{j=1}^{J_i} c_{ij} x'_{ij}\beta\right)}{\sum_{d_j \in \mathcal{B}_i} \exp\left(\sum_{j=1}^{J_i} d_j x'_{ij}\beta\right)}, \tag{2}$$

where $c_{ij} \in \{0,1\}$ and $\mathcal{B}_i = \left\{(d_1, ..., d_{J_i}) : d_j \in \{0,1\}, \sum_{j=1}^{J_i} d_j = \sum_{j=1}^{J_i} c_{ij}\right\}$.

The traditional approach is to estimate β by maximizing a conditional likelihood function based on (2). This, however, would be extremely computationally intensive. For example, our sample of women with a high school degree or less contains a group (state-month combination) with 36,071 observations of whom 18,408 reported working. This means that for this group, the number of terms in the denominator of (2) is $\binom{36{,}071}{18{,}408}$, which is of the order of magnitude of $10^{10{,}852}$. This makes estimation based on the likelihood function in (2) infeasible. To overcome this, we note that for any two individuals in group i, j_1 and j_2,

$$P\left(y_{ij_1} = 1 | \, x_{ij_1} x_{ij_2}, y_{ij_1} + y_{ij_2} = 1\right) = \frac{\exp\left(\left(x_{ij_1} - x_{ij_2}\right)' \beta\right)}{1 + \exp\left(\left(x_{ij_1} - x_{ij_2}\right)' \beta\right)}.$$

This can be used to form a likelihood function for a pair of observations within each group, and combining these pairs would lead to a pseudo-likelihood function. In principle, one could use all pairs of (j_1, j_2) in a group, but this can again be computationally demanding. We therefore pair each observation for whom $y_{ij_1} = 1$ with a randomly chosen observation, ij_2, with $y_{ij_2} = 0$ in the same group. We also pair each observation for whom $y_{ij_1} = 0$ with a randomly chosen observation with $y_{ij_2} = 1$ in the same group. The estimator of β is then based on maximizing the pseudo log-likelihood function

$$\sum_{i=1}^{n} \sum_{j_1=1}^{J_i} \log \frac{\exp\left(y_{ij_1} \left(x_{ij_1} - x_{ij_2}\right)' b\right)}{1 + \exp\left(\left(x_{ij_1} - x_{ij_2}\right)' b\right)}$$

over b. Statistically, this estimator is an extremum estimator (M-estimator) based on the n groups. Assuming independence across groups, consistency of the estimator therefore follows from the fact that each term corresponds to a conditional likelihood, and its asymptotic distribution follows from the standard expression for extremum estimators. See, for example, Amemiya (1985). In particular, the estimator will be asymptotically normally distributed with an asymptotic variance that can be estimated by sample analogs.

Table 3 displays the estimated coefficient in a fixed effects logit version of the model in Table 6 of the paper.[2] The relative magnitudes and the statistical significance of the coefficients in Table 3 are not dramatically different from those of the linear probability model. On the other hand, the coefficients from the logit model are not directly comparable to those of the linear probability

[2] The standard errors reported in Table 3 account for the group structure, but they are not clustered at the household level. The estimation also does not employ sampling weights.

model. In a cross sectional setting, one would therefore convert the logit parameters to average marginal effects. This is not possible with a fixed effects logit approach because the average marginal effect depends on the true conditional probability that y_{ij} is 1, which in turn depends on the distribution of the fixed effects conditional on the explanatory variables. To overcome this, we consider a hypothetical heterogeneous population of East Asians in which the probability of working prior to the pandemic is uniformly distributed between 50% and 90%[3]. The estimated average effects of the pandemic on the probability of working (relative to the effect for Whites with the same education and gender) for this population are declines in the probability of working by 19.1, 14.3, 1.2, 18.9, 11.0, and 1.0 percentage points across the six combinations of education and gender. These are quite similar to the results for the linear probability model in Table 6 of the paper. As a result, we do not further investigate alternatives to the linear probability model.

2.2 Comparison to the 2008 Recession

It is difficult to know the mechanisms behind the dramatic impact of the pandemic on Asians, especially East Asians, with lower educational attainment. One possibility is that this is a typical feature of economic downturns. In order to investigate this possibility, we estimate the same model as in Table 2 of the paper using data from 2006 through 2011, with the "Crisis" variable defined as a dummy variable for the crisis years 2008 through 2011. The results are presented in Table 4.[4] They suggest that there is generally no differential in the impact of the recession between Asians and Whites. This is in sharp contrast to the COVID-19 crisis. The maximum T-statistic of the 18 coefficients that measure the differential Asian-versus-White impact of the crisis is around 2.5, and a joint test of significance across all 6 combinations of education and gender yields a chi-square test statistic of around 14. Compared to a chi-square distribution with 18 degrees of freedom, this is statistically insignificant.

3 Response to Issues Brought Up In the Editorial Process

This section addresses two specific issues that were brought up in the editorial process.

The first issue is whether the results are sensitive to the specification of the dependence of age as being quadratic and whether the number of children matters (as opposed the presence of any children). To address this, we re-estimated the model reported in Table 6 with a fourth degree polynomial in age and with dummy variables for the number of children and the number of children less than 5. Of the 48 coefficients reported in Table 6 of the paper, 35 were unchanged while twelve coefficients changed by one in the third decimal point. One estimate changed by two

[3] The overall probability of working is roughly 70% and we therefore report an average marginal effect across individuals with probabilities between 50% and 90%.

[4] Here, we allow for different effects for East Asians and South East Asians since the sample sizes are much larger than what we consider for the COVID-19 pandemic.

Supplementary Material Table 3: Logit Model for the Probability of Working with Controls for Demographics and Time-varying Labor Market Conditions

	Men HS or less	Men Some Coll.	Men College+	Women HS or less	Women Some Coll.	Women College+
Black*Cr2	-0.094	-0.166**	-0.336***	-0.135**	-0.160**	0.027
	(0.067)	(0.081)	(0.110)	(0.057)	(0.072)	(0.089)
Hispanic*Cr2	-0.388***	-0.294***	-0.168	-0.149**	-0.032	-0.174**
	(0.071)	(0.092)	(0.105)	(0.069)	(0.088)	(0.080)
AsianEast*Cr2	-0.877***	-0.669***	-0.061	-0.871***	-0.521***	-0.051
	(0.147)	(0.184)	(0.113)	(0.118)	(0.164)	(0.104)
AsianOther*Cr2	-0.736***	-0.448**	-0.031	0.129	-0.214	0.288***
	(0.160)	(0.188)	(0.144)	(0.134)	(0.202)	(0.104)
Black*Cr3	-0.092	-0.101	-0.209**	-0.199***	-0.304***	0.035
	(0.080)	(0.087)	(0.102)	(0.066)	(0.070)	(0.083)
Hispanic*Cr3	-0.257***	-0.025	-0.106	-0.061	-0.202***	-0.032
	(0.077)	(0.100)	(0.105)	(0.078)	(0.071)	(0.076)
AsianEast*Cr3	-0.475***	0.031	0.131	-0.288**	-0.296*	-0.001
	(0.161)	(0.156)	(0.119)	(0.114)	(0.155)	(0.102)
AsianOther*Cr3	-0.029	-0.396**	0.143	0.103	0.069	-0.065
	(0.186)	(0.190)	(0.118)	(0.161)	(0.227)	(0.132)
Observations	544,672	358,629	492,165	489,348	419,192	597,483

The table show the coefficient in the logit model with group specific effects. The dependent variable is working, and the sample is restricted to those who had a job in the previous month. The control variables not reported are: age, age^2, marital status, presence of a child, presence of a child under 5, interactions between calendar quarter and ethnicity as well as their main effects, interactions between ethnicity and Cr4 and Cr5, fixed effects for each combination of state and month starting in April 2020, and fixed effects for occupation (using the 2010 Census occupation coding scheme) and each combination of lagged occupation and month starting in April 2020. Cr2, Cr3, Cr4, and Cr5 refer to the second quarter of 2020 through the first quarter of 2021. The data is from IPUMS CPS and covers individuals aged 25-65 over the period between April 2017 and March 2021. Robust standard errors are clustered at the household level.

in the third decimal. We therefore conclude that the results are not sensitive to the functional form for age or for the number of children.

The second issue is whether it is more relevant to control for individual or occupation fixed effects. In the paper, we include fixed effects for the interactions between occupation and month-dummies (after the onset of the crisis) because we expected this to be economically more relevant than individual specific fixed effects. It is likely that observations for a given individual in different time periods will be correlated, and the results reported in the paper attempt to account for this by calculating standard errors that are clustered at the household level. To investigate the relative importance of the two types of fixed effects, we re-estimated the model in Table 6 of the paper using no fixed effects and using individual fixed effects (without sampling weights because this can be difficult to deal with in a traditional panel data setting if the weights vary over time).

Supplementary Material Table 4: The Probability of Working with Controls for Demographics and Time-varying Labor Market Conditions, 2008 Recession

	Men HS or less	Men Some Coll.	Men College+	Women HS or less	Women Some Coll.	Women College+
Black*Crisis	-0.005	-0.019**	-0.027***	-0.019***	-0.016**	-0.036***
	(0.007)	(0.008)	(0.008)	(0.007)	(0.007)	(0.007)
Hispanic*Crisis	-0.012**	0.001	-0.009	-0.006	-0.014	-0.006
	(0.005)	(0.007)	(0.007)	(0.007)	(0.009)	(0.010)
AsianE*Crisis	0.038**	-0.010	-0.013	0.003	0.001	-0.011
	(0.019)	(0.025)	(0.011)	(0.019)	(0.025)	(0.014)
AsianSE*Crisis	0.001	0.017	0.003	0.007	0.017	-0.018
	(0.017)	(0.018)	(0.013)	(0.018)	(0.020)	(0.014)
AsianOther*Crisis	-0.008	-0.006	-0.011	-0.000	-0.032	0.004
	(0.020)	(0.018)	(0.009)	(0.022)	(0.026)	(0.015)
Observations	1,042,906	641,817	781,625	1,013,730	773,306	862,519

The dependent variable is working. The control variables not reported are: age, age^2, marital status, presence of a child, presence of a child under 5, interactions between calendar quarter and ethnicity as well as their main effects, and fixed effects for each combination of state and month starting in January 2008. Crisis refers to the quarters starting January 2008. The data is from IPUMS CPS and covers individuals aged 25-65 over the period between January 2006 and December 2011. Robust standard errors are clustered at the household level, and the estimation uses sampling weights.

For comparison, we also re-estimated the model in Table 6 of the paper without weights. The results are given in the three tables below.

It is clear from the estimation results that controlling for the interactions between occupation and the crisis-month-dummies makes a larger difference than controlling for individual fixed effects. (See, for example, the numbers displayed in bold.)

Supplementary Material Table 5: NO FIXED EFFECTS

	Men HS or less	Men Some Coll.	Men College+	Women HS or less	Women Some Coll.	Women College+
Black*Cr2	-0.038***	-0.034**	-0.045***	-0.032**	-0.013	-0.013
	(0.013)	(0.014)	(0.012)	(0.016)	(0.013)	(0.010)
Hispanic*Cr2	-0.032***	-0.010	-0.016*	-0.020	-0.027*	-0.034***
	(0.009)	(0.012)	(0.010)	(0.014)	(0.014)	(0.012)
AsianEast*Cr2	**-0.168*****	-0.070**	-0.028**	**-0.186*****	-0.072**	-0.024**
	(0.031)	(0.028)	(0.011)	(0.034)	(0.032)	(0.012)
AsianOther*Cr2	-0.070**	-0.099***	-0.004	-0.018	-0.112***	0.006
	(0.031)	(0.036)	(0.009)	(0.036)	(0.042)	(0.013)
Black*Cr3	0.002	-0.006	0.000	-0.017	-0.029**	-0.007
	(0.010)	(0.011)	(0.009)	(0.012)	(0.012)	(0.009)
Hispanic*Cr3	-0.009	0.005	0.001	0.012	-0.003	-0.021*
	(0.007)	(0.009)	(0.008)	(0.010)	(0.012)	(0.011)
AsianEast*Cr3	-0.004	-0.033*	-0.001	-0.046**	-0.004	-0.006
	(0.018)	(0.020)	(0.008)	(0.022)	(0.021)	(0.010)
AsianOther*Cr3	0.008	-0.013	0.008	0.030	0.038	0.001
	(0.020)	(0.025)	(0.008)	(0.031)	(0.027)	(0.013)
Observations	268,219	195,225	295,552	180,794	193,398	311,497

The dependent variable is working, and the sample is restricted to those who had a job in the previous month. The control variables not reported are: age, age^2, marital status, presence of a child, presence of a child under 5, interactions between calendar quarter and ethnicity as well as their main effects, fixed effects for each combination of state and month starting in April 2020, and interactions between ethnicity and Cr4 and Cr5. The data is from IPUMS CPS and covers individuals aged 25-65 over the period between April 2017 and March 2021. Robust standard errors are clustered at the household level.

Supplementary Material Table 6: INDIVIDUAL FIXED EFFECTS

	Men HS or less	Men Some Coll.	Men College+	Women HS or less	Women Some Coll.	Women College+
Black*Cr2	-0.054***	-0.042**	-0.051***	-0.039*	-0.034*	-0.010
	(0.018)	(0.019)	(0.016)	(0.021)	(0.018)	(0.013)
Hispanic*Cr2	-0.039***	-0.013	-0.023*	-0.037**	-0.046**	-0.033**
	(0.012)	(0.016)	(0.014)	(0.019)	(0.020)	(0.017)
AsianEast*Cr2	**-0.170*****	-0.097***	-0.011	**-0.213*****	-0.080*	-0.002
	(0.039)	(0.035)	(0.015)	(0.041)	(0.041)	(0.015)
AsianOther*Cr2	-0.079**	-0.099**	-0.005	-0.066	-0.099*	0.006
	(0.040)	(0.049)	(0.011)	(0.046)	(0.051)	(0.018)
Black*Cr3	-0.012	-0.028	-0.001	-0.005	-0.058***	-0.015
	(0.014)	(0.017)	(0.014)	(0.016)	(0.017)	(0.012)
Hispanic*Cr3	0.003	-0.002	0.004	0.024	-0.048***	-0.014
	(0.010)	(0.014)	(0.013)	(0.015)	(0.018)	(0.016)
AsianEast*Cr3	-0.025	-0.015	0.003	-0.030	-0.026	-0.004
	(0.026)	(0.034)	(0.012)	(0.029)	(0.028)	(0.013)
AsianOther*Cr3	0.020	-0.027	0.017	0.007	0.036	0.005
	(0.027)	(0.030)	(0.010)	(0.053)	(0.052)	(0.019)
Observations	268,219	195,225	295,552	180,794	193,398	311,497

The dependent variable is working, and the sample is restricted to those who had a job in the previous month. The control variables not reported are: age, age^2, marital status, presence of a child, presence of a child under 5, interactions between calendar quarter and ethnicity as well as their main effects, fixed effects for each combination of state and month starting in April 2020, interactions between ethnicity and Cr4 and Cr5, and individual fixed effects. The data is from IPUMS CPS and covers individuals aged 25-65 over the period between April 2017 and March 2021. Robust standard errors are clustered at the household level.

Supplementary Material Table 7: FIXED EFFECTS FOR OCCUPATION INTERACTED WITH MONTH (AFTER ONSET OF CRISIS)

	Men HS or less	Men Some Coll.	Men College+	Women HS or less	Women Some Coll.	Women College+
Black*Cr2	-0.025*	-0.008	-0.036***	-0.040**	-0.013	-0.015
	(0.013)	(0.013)	(0.011)	(0.016)	(0.013)	(0.010)
Hispanic*Cr2	-0.024**	-0.008	-0.010	0.003	-0.013	-0.024**
	(0.010)	(0.012)	(0.010)	(0.014)	(0.013)	(0.011)
AsianEast*Cr2	**-0.104***	-0.065**	-0.022**	**-0.099***	-0.041	-0.021*
	(0.032)	(0.027)	(0.011)	(0.032)	(0.028)	(0.011)
AsianOther*Cr2	-0.047	-0.086**	-0.013	-0.021	-0.075**	0.003
	(0.032)	(0.035)	(0.008)	(0.036)	(0.036)	(0.012)
Black*Cr3	0.006	-0.008	0.001	-0.021	-0.030**	-0.011
	(0.010)	(0.012)	(0.009)	(0.013)	(0.012)	(0.009)
Hispanic*Cr3	-0.009	0.003	-0.003	0.011	-0.004	-0.018
	(0.008)	(0.009)	(0.008)	(0.011)	(0.012)	(0.011)
AsianEast*Cr3	-0.010	-0.028	-0.007	-0.044*	-0.012	-0.014
	(0.019)	(0.020)	(0.009)	(0.024)	(0.020)	(0.010)
AsianOther*Cr3	-0.000	-0.018	0.005	0.020	0.040	-0.004
	(0.022)	(0.026)	(0.008)	(0.033)	(0.027)	(0.013)
Observations	268,219	195,225	295,552	180,794	193,398	311,497

The dependent variable is working, and the sample is restricted to those who had a job in the previous month. The control variables not reported are: age, age^2, marital status, presence of a child, presence of a child under 5, interactions between calendar quarter and ethnicity as well as their main effects, fixed effects for each combination of state and month starting in April 2020, interactions between ethnicity and Cr4 and Cr5, and fixed effects for occupation (using the 2010 Census occupation coding scheme) and each combination of lagged occupation and month starting in April 2020. Cr2, Cr3, Cr4, and Cr5 refer to the second quarter of 2020 through the first quarter of 2021. The data is from IPUMS CPS and covers individuals aged 25-65 over the period between April 2017 and March 2021. Robust standard errors are clustered at the household level.

References

Amemiya T (1985) Advanced Econometrics. Harvard University Press

Arellano M, Hahn J (2007) Understanding bias in nonlinear panel models: Some recent developments. In: Advances in Economics and Econometrics, Ninth World Congress, Cambridge University Press

Lancaster T (2000) The incidental parameter problem since 1948. Journal of Econometrics 95(2):391–413, DOI https://doi.org/10.1016/S0304-4076(99)00044-5, URL https://www.sciencedirect.com/science/article/pii/S0304407699000445

Neyman J, Scott EL (1948) Consistent estimates based on partially consistent observations. Econometrica 16:1–32

Rasch G (1960) Probabilistic Models for Some Intelligence and Attainment Tests. Denmarks Pædagogiske Institut, Copenhagen

Rasch G (1961) On general laws and the meaning of measurement in psychology. In: Proceedings of the Fourth Berkeley Symposium on Mathematical Statistics and Probability, Volume 4: Contributions to Biology and Problems of Medicine, University of California Press, Berkeley, Calif., pp 321–333, URL https://projecteuclid.org/euclid.bsmsp/1200512895
